# Self-efficacy and (vocational) interest in technology and design: an empirical study in seventh and eighth-grade classrooms

**DOI:** 10.1007/s10798-018-9475-y

**Published:** 2018-11-07

**Authors:** Karin Güdel, Anni Heitzmann, Andreas Müller

**Affiliations:** 1 Department of Teacher Education, Secondary 1 Level, University of Applied Sciences, Clarastrasse 57, 4058 Basel, Switzerland; 2 Department of Teacher Education, University of Applied Sciences, Niederriedweg 110, 3019 Bern, Switzerland; 3 Faculty of Science, Physics Department, and Institute of Teacher Education, Pavillon d’Uni Mail (IUFE), University of Geneva, Boulevard du Pont d’Arve 40, 1211 Geneva, Switzerland

**Keywords:** Technology education, Science, Design process, Interest, Self-efficacy, Vocational interest

## Abstract

The aim of this article is to report the results of an empirical study on adolescents’ interest, self-efficacy, and vocational interest in technology and design. Following the expectancy-value model, we wanted to know how context-specific interest in technology and perceived self-efficacy in solving technical tasks are developed at lower secondary-school level and how they predict vocational interest in technology. These personal-trait variables were operationalized in an online survey conducted among 480 students (seventh and eighth grade) in Northwestern Switzerland. Quantitative analyses showed that interest, self-efficacy, and vocational interest vary with respect to theoretical, practical, and creative activities. Moreover, there were marked gender differences in interest and self-efficacy, especially regarding “Using and repairing technical tools” and “Understanding technological processes.” No gender differences could be found in “Designing in the context of sustainability,” however. Interest, self-efficacy, and vocational interest correlate very highly, but self-efficacy can predict vocational interest in technology better than interest. These results are discussed in the context of recent developments (e.g. STEM initiatives) in the field of technology education worldwide and in particular in Switzerland. According to our analyses and the current discussions about more STEM education and technically skilled teaching staff, schools should provide all students with opportunities to deal with technology, thus enabling them to make manifold experiences in theoretical, practical, creative, and critical ways from early childhood until career choice. At present, this does not seem to be sufficiently the case because otherwise girls would probably not have such negative perceptions of their own abilities.

## Introduction

The promotion of science and technology education is often argued for with the lack of professionals in this field. In the past 20 years, a lot of STEM initiatives have been implemented at both primary-school and lower secondary-school level, all of which pursuing the aim of attracting more young people’s, especially girls’, interest in technology. The mere existence of such initiatives does not by itself guarantee a positive influence on the attitudes of adolescents towards technology, of course, but the actual effects of these programs on interests and career choices are difficult to measure, and they have only seldom been investigated so far, neither by the program leaders themselves nor by external experts.

Against this background, one challenge facing engineering and technology educators is how to introduce and teach technology in a way that appeals to a majority of the students and gives them the opportunity to make positive experiences with the subject area. This challenge involves motivational and cognitive aspects that have an impact on student learning. Solving an ill-structured problem such as an engineering design task, for instance, requires a wide range of cognitive processes (Lawanto and Stewardson 2013). A recent study showed that experiences with a holistic approach to the teaching of design processes (inventing, developing, understanding, designing, constructing, and evaluating artefacts/products) do not differ between girls and boys in terms of interest and learning outcomes (Guedel 2014). Nevertheless, interest in taking up a job that involves the same activities (inventing, developing, etc.) tends to be very stereotypical, with boys being much more interested than girls. What is the reason for this?

Taking the expectancy-value model (Eccles and Wigfield 2002), which explains how motivational factors and beliefs affect achievement and achievement-related choices, as a theoretical basis, we are currently trying to gain an in-depth insight into how interest and self-efficacy affect career interest in the field of technology. The main variables of the model are “Success,” “Ability beliefs” (e.g. self-efficacy), and “Task value” (e.g. interest). For the purposes of this paper, we investigated these variables in the context of specific activities and problem-solving tasks related to design processes in general and with regard to possible future careers. The results of our analyses are supposed to make not only educators but also politicians and gender experts aware of where to take action in order to establish equal opportunities in the field of technology. As long as the beliefs of girls in their abilities in technology-specific tasks remain as low as they are at present, the impact of STEM or technology promotion programs must be questioned.

## Theoretical framework

In this section, we introduce the two personal-trait variables “Individual interest” and “Self-efficacy” as well as a simplified model derived from the expectancy-value model by Eccles and Wigfield (2002). Furthermore, we report a selection of pertinent findings from empirical studies in the field of technology and design education, which leads to three research questions.

### Individual interest

A generally accepted theory in educational psychology holds that that interest is always directed towards an object, that is for example to specific topics, knowledge domains, activities, or goals (Krapp 1998; Krapp and Prenzel 2011). This implies that one cannot simply be interested but that one is always interested *in something* (see Gardner 1996). Moreover, interest determines the relationship between the person and the object of interest. Different types of appeal lead to an interest-oriented occupation with an object. Interest that is aroused by a cognitive, emotional, or value-related appeal in a specific situation is called “situational interest.” If an interest already exists because the object poses a cognitive challenge or arouses positive emotions, or because dealing with the object is regarded as a potential benefit, this is referred to as an individual interest (Krapp and Prenzel 2011). For operationalizing interest, cognitive, emotional, and value-related facets have to be taken into account (Krapp and Prenzel 2011). As they may vary with the same overall interests, also different activities, contents, and contexts pertaining to the object need be distinguished (Hoffmann et al. 1998; Todt 1995). For these reasons and because interests become more specific with increasing age (Daniels 2008; Krapp and Prenzel 2011), it is essential to go beyond global assessments like those of TIMSS and PISA (see, e.g., Martin et al. 2012; OECD 2007) where interests are considered without any particular focus.

### Self-efficacy

A feeling that is closely linked to the feeling of competence, which is one of the basic psychological needs involved in the development of interests (Deci and Ryan 1993), is the feeling of self-efficacy (Bandura 1993, 1997, 2000). Self-efficacy is defined as “beliefs in one’s capabilities to organize and execute courses of action required to produce given attainments” (Bandura 1997, p. 3). The literature usually draws a distinction between a relatively stable general self-efficacy trait (Jerusalem and Hopf 2002; Schwarzer and Jerusalem 2002) and domain- or situation-specific self-efficacy that sensitively responds to personal developments and influences of the environment. The latter has often been used as an indicator of changes induced in teaching interventions (Bandura 1997; Zimmerman 2000).

### Expectancy-value theory

**Fig. 1:**
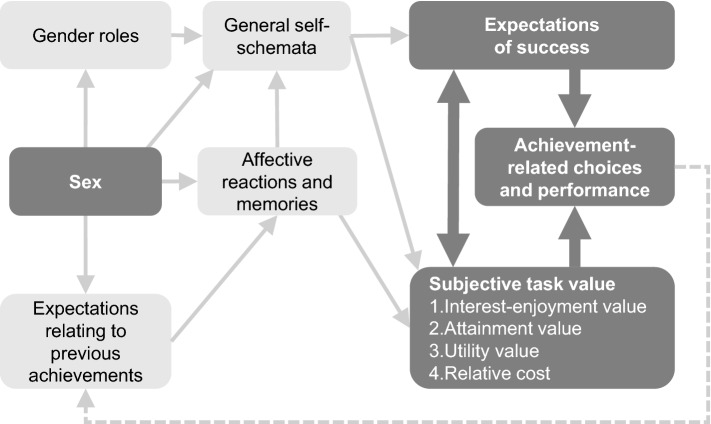
Model of indirect influences (light, thin arrows) of sex on expectations of success (e.g. self-efficacy), subjective task value (e.g. interest), achievement-related choices and performance (e.g. career choice) and direct influences (dark, bold arrows) among these three variables (based on the expectancy-value model by Eccles and Wigfield 2002, p. 119)

According to the expectancy-value model proposed by Eccles and Wigfield (2002), expectations of success and subjective task values can be assumed to influence achievement-related choices and performance in a direct way (see Fig. 1). Expectations of success can be understood as an individuals’ beliefs about how well he or she will be able to cope with a given task, either in the immediate or long-term future. Such beliefs can be measured in a manner that analogous to measures of Bandura’s (1997) personal efficacy expectations. In the context of teaching and learning, Eccles and Wigfield (2002, p. 119) divide the subjective value attributed to a task or a topic area into four components:

1. “interest-enjoyment value” (interest, task as a source of fun);
2. “attainment value” (importance, identification with the subject and school in general);
3. “utility value” (benefits regarding short-term and long-term objectives);
4. “relative cost” (opportunity costs).

The model assigns self-efficacy to the expectations-of-success component (Eccles and Wigfield 2002, pp. 110–111) while the various components of interest correspond to the subjective value of the task (Eccles and Wigfield 2002, pp. 114–115). The value-related component of interest can be assigned to the attainment value and the emotion-related component of interest to the intrinsic value. According to the model, sex and the gender-specific self-concept influence self-efficacy and interest via an individual’s perceptions, goals, and general self-schemata. These, in turn, influence career choice.

In the context of technology education, not many researchers have looked at the correlations and influences between interest, self-efficacy, and career choice or other constructs explained by Eccles and Wigfield (2002) although they predict choices of tasks and future jobs. Studies by Lawanto et al. (2012) as well as Lawanto and Stewardson (2013) were able to identify correlations between interest and expectations of success in engineering design tasks but their findings say nothing about causal relations.

### (Vocational) interest and self-efficacy in the field of technology

#### Interest in technology

In the field of technology, the theory of the object specificity of individual interest (dependence on activities, contexts, and contents) explained above has not been applied yet, except for interests in some domains like electrical engineering or environmental technology. These studies found large differences both between different content areas and between the sexes (e.g. Acatech and VDI 2009). In studies dealing with situational interest in technical teaching, the aspects of practical work (hands-on) and constructing and building were identified as a cause of interest (see Dohn 2013). The German MoMoTech study (Acatech 2011, p. 95) recommended an inductive approach in upper secondary education in order to transform situational interest into individual interest and technological competence. According to such inductive approaches, the students first deal with tools and materials in a practical way, which can then lead to a skilled handling and understanding of complex technical equipment like microscopes, telescopes, measuring instruments, and computers. Interest scales that measure scientific and technical interest are often limited to the epistemic component of interest, however, focusing on the subjectively felt need to “know more” or “learn more” (Martin et al. 2012; OECD 2007).

In contrast to the natural sciences, where scientific methods of gaining (new) knowledge lie at the core of the disciplines, the innovations in technology relate to a realistic and true-to-life form of problem solving. As the findings of a study conducted by Acatech and VDI (2009) indicate, problem solving in real-life contexts can be of interest to young people. Boerlin et al. (2014) showed in their survey in Switzerland that girls are somewhat interested in crafts whereas physics is considered to be the least interesting subject. Interest in activities that form part of a design process, which could belong to physics as well as to craft-related subjects, has never been investigated, however. It is for this reason that our study focuses on technology-specific interests and measures them with respect to different contexts and different activities within a design process.

#### Vocational interest in technology

If the research focus is on vocational interest in technology, further factors besides general interest in technology have to be taken into account. According to Gottfredson (2002), the common public representation of the sexes is the most important criterion as regards career choice. A decision for an “atypical” profession requires a lot of self-confidence and sometimes demands considerable sacrifices. In the professional field of technology, such gender-related obstacles are particularly pronounced (Herzog et al. 2006). In many societies, technology-related professions are traditionally dominated by men—with the exception of the fields of (para)medical treatments and creative arts. Thus, in view of the widely discussed problem of lacking workforce in the STEM area (Gehrig et al. 2010), it is important to understand better why an individual decides for or against a technical profession and why only a few women are interested in a career in this area.

The reasons that account for whether or not to take up a career in technology are manifold and largely coincide with the reasons for a high or low interest in technology. A positive or a negative basic attitude towards technology usually evolves already at an early stage of the development of gender roles (Ziefle and Jakobs 2009). In a first phase, preferences are shaped through the reinforcement from parents and other reference persons and later on through the emulation of role models that are typically embodied by men (Rost 2001, lemma “Geschlechterunterschiede”). Often this means that the development of scientific and technical interests is less encouraged in girls than in boys (Pfenning and Schulz 2012). What is also not to be neglected in this context is that technical interest is frequently influenced by (experienced) Pygmalion-effects (Rosenthal and Jacobson 1968) in the classroom: at lower secondary-school level, high technical and mathematical skills are often attributed to boys whereas girls are said to be competent in languages, even if there is no difference between the actual abilities of boys and girls (Acatech and VDI 2009). This finding is in line with the assumptions of expectancy-value theory holding that not only interest (intrinsic motivation) influences vocational choice but also the belief in one’s own abilities (expectations of success). The fact that boys and girls often differ considerably in this respect contributes substantially to explaining the gender gap in the field of technology.

#### Technology-specific self-efficacy

Self-efficacy in dealing with technology corresponds to the perception of one’s own ability to carry through technical actions successfully, to deal with technical problems, and to solve them adequately. It is tightly related to the beliefs of competence and control. Recent research findings concerning subjective expectations in terms of one’s own abilities to be successful in coping with technical and/or scientific tasks and learning can be summarized as follows: technology-specific self-efficacy differs markedly between different areas of activities. In particular, self-assessments of one’s own skills in dealing with everyday technology on a *practical* plane are relatively positive and get even more positive with age (Ziefle and Jakobs 2009) whereas they are much less positive as regards *theoretical* aspects of technology. Moreover, related studies in science education indicate that the latter type of assessment even becomes more negative the course of schooling at secondary level I (Acatech and VDI 2009; Körner et al. 2012; Martin et al. 2008). Gender differences in self-efficacy were found in all investigated subfields, except for computer knowledge. In summary, girls, regardless of their actual abilities, systematically feel less competent in dealing with technology and understanding technical problems than boys.

### Technology education in different subjects

The new Swiss curriculum “Lehrplan 21” (D-EDK 2016) makes references to technology education in four different subjects: “Technical and Textile Design,” “Nature and Technology,” “ICT and Media,” and “Economy, Labour and Housekeeping.” The decisive question is whether the intended interconnections between the different subjects, which are explicitly mentioned in Lehrplan 21, will indeed be adopted and put into effect in practice. If so, this could be a promising starting point for strengthening technology education in Switzerland in the next few years.

**Fig. 2:**
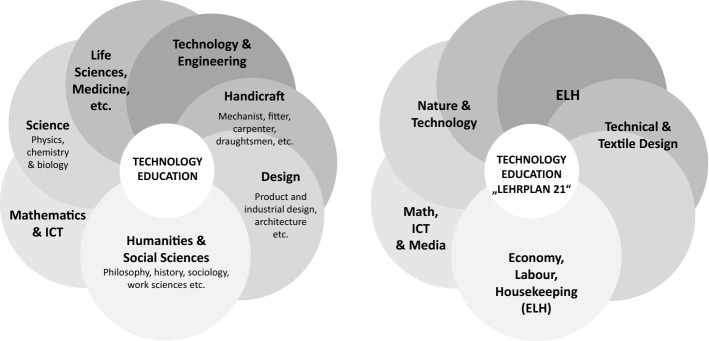
Illustration of different domains of technology education in tertiary education and vocational training (left) and corresponding subjects relating to technology education at school according to “Lehrplan 21” (right) (Guedel and Heitzmann 2016)

Primary school, where “Nature, Man, Society” is taught as an integral subject, would theoretically provide ideal conditions for discussing and approaching technology from a variety of perspectives, for example along the lines of the German concept of “multi-perspective technology education.” In reality, however, teachers do often not dare to deal with technical problems because on one hand they do not feel competent enough and on the other hand they do not have enough time for practical activities. Besides, the infrastructure of the classrooms does usually not allow handicraft and experimental and technology-oriented teaching. At lower secondary-school level, the combination subject “Nature, Man, Society” is split into four distinct subject areas, two of which with strong connections to technology: “Nature and Technology” and “Economy, Labour, and Housekeeping.” A comparison between the different domains of technology education in tertiary and vocational education and the corresponding school subjects relating to technology education according to Lehrplan 21 reveals that most domains are represented by a school subject except for “Technology and Engineering” (see Fig. 2). In the curriculum for upper secondary school, the subject “Technical and Textile Design” has been completely abolished, and science is taught separately in the three traditional, distinct main subjects “Physics,” “Chemistry,” and “Biology.” Thus, engineers and technicians are not involved in compulsory schooling in Switzerland, and the same applies to current teacher education programs.

One major goal of politically motivated STEM initiatives (in German “MINT”), which are often justified by invoking economic arguments (e.g. lack of skilled work force), is to strengthen technology and engineering. With the intention of achieving this aim, the Swiss curriculum designers have “enriched” education in natural sciences with subject matter from technology (“Nature and Technology”), but at the same time they have cut “soft” subjects like arts and handicraft (fewer prescribed classes per week, less significance in teacher education, etc.). In sum, the scientific approach to technology is now promoted while the technical-creative approach has been considerably weakened. With the introduction of the new subject “Economy, Labour, and Housekeeping,” at least some social and economic aspects of technology education appear in the new curriculum. In view of this situation and the fact that young people are permanently surrounded and increasingly influenced by technology, Swiss schools and teacher education are undeniably required to strengthen technology and engineering by integrating them in a multifaceted way into *all* technology-related subjects and to do so from the very start of primary school until the end of secondary school when adolescents have to make their career choices.

### Research questions

According to the theoretical outline, the research findings and the situation in Switzerland presented above, it was of interest for us to analyze technology-related interests of adolescents by distinguishing between different areas of life (school, leisure time, and future job) and specific activities within a design process in general or in potential jobs. Moreover, interest and self-efficacy are personal-trait variables that are connected to each other and both influence vocational interests. Taking these premises into account, the three research questions to be answered in this paper are the following:

1. How do technology-related interests of girls and boys differ between different contexts and different activities in a design process?
2. How does self-efficacy in technical tasks as perceived by girls and boys differ between different tasks (irrespective of the content) within the design process?
3. Can vocational interest in the field of technology and design be predicted by interest in technology and self-efficacy in technical tasks?

## Materials and methods

The data for this study were gathered within a larger project, an intervention study, about interest and interest development (Heitzmann 2010). It was conducted in seventh-grade and eighth-grade classrooms from lower secondary-level schools in North-Western Switzerland (International Standard Classification of Education Level 2; Bfs 2008). The present work focuses on the influences of gender and self-efficacy on several facets of interest, as formulated in the research questions in “Research questions” section. This study is built on data obtained at the beginning of the project prior to an intervention (not described here). More detailed information on the intervention and a comprehensive presentation of the results of the and pre-, post- and follow-up tests can be found in Guedel (2014) and Guedel et al. (2015).

### Instruments and sample

For measuring relatively stable individual interests and self-efficacy, self-report surveys have proved to be a useful data source and have therefore been used in many studies (e.g. Krapp and Prenzel 2011; Renninger and Hidi 2011). It is important, however, that the operationalization is carried out on the basis of clearly defined psychological constructs and that a variety of dimensions and facets of interest are covered.

In our study, we measured the participants’ individual interest in technical activities during a design process, their feelings of self-efficacy in solving technical tasks, and their vocational interests by means of a standardized questionnaire. It contained scales that had previously been tested and validated in large-scale assessments as well as scales whose reliability and validity had to be ascertained beforehand because of adaptations to the subject of technology. A pilot study using these scales was carried out with 102 pupils. A description of this pilot study and the scales is provided in Guedel (2014). All scales presented in this paper were 4-point Likert scales. The adapted, final version of the questionnaire was individually completed by 483 students, mostly online but in a few exceptional cases in a paper–pencil format.

The participants were seventh and eighth graders from all three academic tracks (I, II, III) of lower secondary school in Northwestern Switzerland. The sample was roughly equally distributed between girls (48%) and boys (52%), grades (7th grade: 45%; 8th grade: 55%) and the academic tracks (Track I: 44%; Track II: 34%; Track III: 22%). Since the track with the highest requirements (III) may seem to be underrepresented, it has to be noted that the actual population of this track is smaller than the populations of the low and the medium track (I and II).

#### General and specific interest in the context of school, leisure time, and future job

General interest in technology in the contexts of school, leisure time, and future jobs was measured with a total of 17 items; sample items of each context are as follows:

- “In my leisure time, I have often worked with technology in the last 6 months” (scale: “Interest in technology in leisure time”).
- “At school, I find it useful to learn about technology” (scale: “Interest in technology at school”).
- “I would be happy to have something to do with technology in my future job” (scale: “Interest in technology in the future job”).

According to international studies (OECD 2007; Sjøberg and Schreiner 2010), interest in working in the field of technology is usually not high, especially in western countries. By analyzing the data in terms of the question concerning the students’ interest in conducting specific activities in their future job, differences and correlations between doing these activities within and without a given context could be explored:

- “In my future job, I would like to construct and produce technical devices myself” (scale: “Interest in technology with regard to specific activities in the future job”).

#### Specific interest in activities within the design process

Interest in activities that are part of the design process was assessed with a total of 25 items in six categories: “Understanding and evaluating,” “Inventing, developing and building,” “Planning and designing,” “Using technical tools,” “Designing eco-friendly product,” and “Designing a product in a team.” The categories had been derived from the areas of technological skills as defined by VDI (2007) and the approach “Explicit, Reflective Technology Education” (Guedel 2014) while the development of the items was in line with the German “Interessensstruktur-Test” (Bergmann and Eder 2005). Some sample are listed below:

I like …/I would like to, if I had the chance to …

- working/work with machines and other technical tools (scale: “Using and repairing”).
- making/make sketches of new products (scale: “Planning and designing”).
- reading/read newspaper articles covering technological issues (scale: “Understanding, explaining and evaluating”).
- developing/develop ideas for new (eco-friendly) products (in a team)” (scales: “Inventing, developing and building”; “Designing eco-friendly product”, “Team”).

#### Self-efficacy in solving technical tasks

In the context of technology education, situational self-efficacy (Schwarzer and Jerusalem 2002) in solving technical tasks is of special interest. Therefore, we identified 19 different tasks and formulated items in accordance with the scale of general self-efficacy proposed by Schwarzer and Jerusalem (2002). Sample items are the following:

- “I’m confident that I can fix a dripping tap myself” (scale: “Using and repairing”).
- “If I make an effort, I can explain technical issues without difficulty” (scale: “Understanding and explaining).
- “Even if I am under time pressure, I can draw very accurate sketches and plans” (scale: “Planning and designing).
- “If my handicraft teacher shows us how to do something, I’m convinced that I will succeed in doing the task myself” (scale: “Craft-related tasks”).

### Data analysis

#### Significance tests and effect sizes

Differences between reliable scales were tested with reference to the research questions. Although the Kolmogorov–Smirnov test showed a significant deviation from the normal distribution for all scales, *t* tests were carried out. The reason for this procedure is that according to Bortz (2006) and other reference works in statistics, many statistical methods are relatively robust with respect to the violation of the conditions in roughly equally sized samples with *n* > 30. Nevertheless, the results were, as far as possible, additionally controlled by distribution-free, that is nonparametric tests (Kruskal–Wallis test and Mann–Whitney U test). In most cases, the significance level was 0.01, in some cases also 0.05. Consequently, the probability of a type I error amounted to a maximum of 1–5%. Effect sizes are reported as Cohen’s *d* using the pooled standard deviation (*d *=(*M*_1_ −* M*_2_)/*SD*_*p*_), and interpreted in accordance with the usual effect-size levels as small (0.2 < *d* < 0.5), medium (0.5 ≤ *d* < 0.8) or large (0.8 ≤ *d*) (Cohen 1988).

#### Principal component and reliability analysis

Before the reliability of two dimensions of the questionnaires (interest and self-efficacy) could be tested, we had to ensure that the theoretically derived dimensions were validated psychological constructs. For this purpose and for reducing the data, we conducted principal component analyses (see Backhaus et al. 2011). As a prerequesite, the conditions of applicabilty were checked (Bühner 2006, pp. 207): KMO coefficient > 0.60; MSA coefficient > 0.60; Bartlett significance; eigenvalue > 1; factor loading (Bortz 2006, p. 534): factors with four or more loadings > 0.60, factors with ten or more loadings > 0.40).

Because the components were not independent, we chose a Promax rotation instead of Varimax (see Bühner 2006, Chapter 5). Details concerning the three principal component analyses can be found in Guedel (2014) and in the Appendix. On the whole, the theoretically derived categories could be matched with the factor analysis.

Reliability in terms of consistency was assessed by means of Cronbach’s alpha (α) for one-dimensional scales (or factors). Scales with α > 0.7 were considered to be sufficiently reliable, although 0.7 is deemed to be a low value whereas 0.8 is regarded as a medium and 0.9 as a high value (see Bühner 2006).

#### Structural equation modelling

**Fig. 3:**
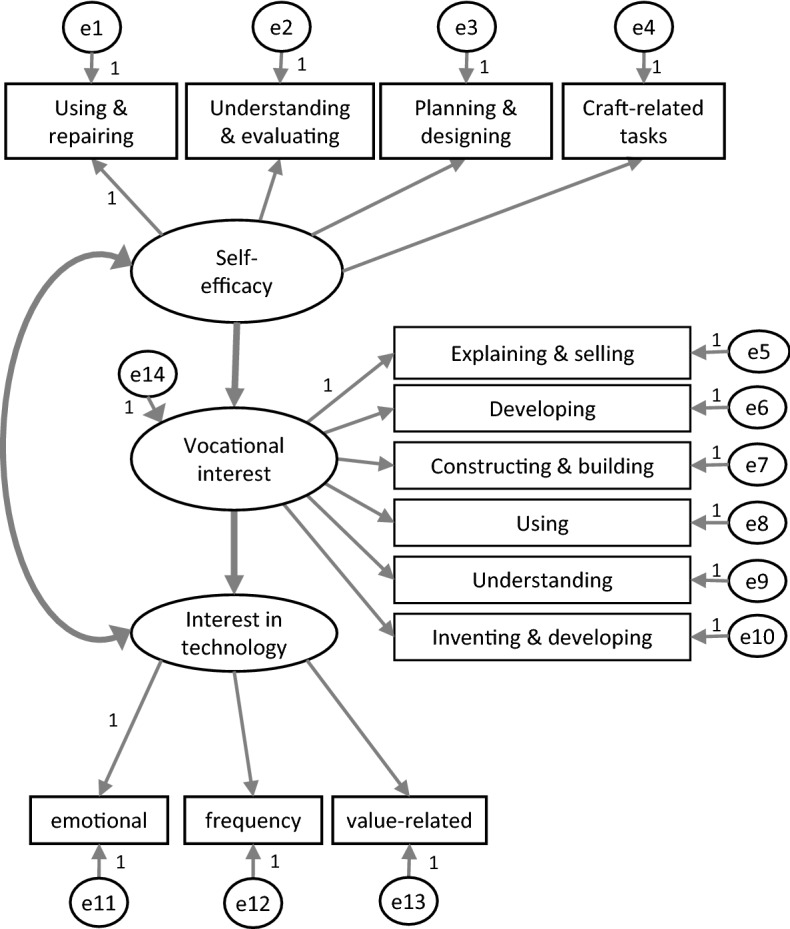
Structural equation model with “Self-efficacy” and “Interest” as independent (endogenous) variables and “Vocational interest” as a dependent (exogenous) variable. The measured variables are represented in boxes (measurement model), the latent variables in ovals (structural/causal model). The e’s in the circles represent the error terms

In order to examine the joint influence of interest in technology and self-efficacy in technical tasks on vocational interest in technology, we made use of structural equation modelling, availing ourselves of the software “Amos 7” (Airbuckle 2007). The main elements of the model consist in three variables that are not directly determined by the questionnaire. These latent variables, represented by ovals in Fig. 3, are associated with at least three manifest scales or single items (represented in boxes in Fig. 3) that were actually measured. The assumption behind this is that the scales or items and their correlations can be explained by a non-observable background variable.

The relationships and influences between the latent variables are presented in structural or causal models while the explanation of the manifest variables through the individual latent variables is called a measurement model. Together they form the structural equation model of the variables in question (Backhaus et al. 2011; Hair et al. 1998). Note that for the calculation of such models, all endogenous variables (latent and manifest variables) that are influenced by another variable must be associated with a possible error, which is represented by small circles (e1–e14) in Fig. 3 and indicates the proportion of variance that is not caused by the latent variable. It is likely that there are correlations between these errors because content-related scales are quite interconnected. In the model, they are represented as covariances, but only significant covariances (< 0.01) were included. Building on theoretical considerations, we were particularly interested in the connections between the two independent (endogenous) variables “Interest” and “Self-efficacy” and the dependent variable “Vocational interest” (represented as bold arrows in Fig. 3). They largely correspond to the dependencies to be examined between the manifest variables. Thus, interest in technology and technology-specific self-efficacy influence vocational interest, and there is a reciprocal relationship between interest and self-efficacy.

## Results

### Research question 1: general and specific interest

The following section presents the results for research question 1: *How do technology*-*specific interests of girls and boys differ between different contexts and different activities within a design process?*

#### General interest in the contexts of leisure time, school, and future job

With 4-point Likert scales the mean values of the items range from 2.11 (“I would like to invent and develop technical devices myself in my future job”) to 3.07 (“In my spare time, I want to have to do with technology as little as possible”, minus pole). In general, the responses vary widely between much and little interest. Making use of principal component analyses, we could extract three factors from a total of 15 items (KMO = 0.89; MSA coefficients > 0.84; see “Appendix 1”). These factors explained 64% of the total variance. The factors can be characterized in terms of their content and indicate a specific context of interest in technology (leisure time, school, future job). The differences between the mean values of the scales summarized in Table 1 show that interest in technology in the context of leisure time (*M* = 2.88) is more pronounced than interest in the context of school (*M* = 2.60) and future job (*M* = 2.26). Furthermore, there are significant differences between girls and boys in all four scales, with boys being much more interested than girls (*d* ≥ 0.5). This gender gap is especially large with respect to specific activities in context of the future job (*d* = 1.2).

**Table 1:** Mean values and gender differences regarding interest in technology in different contexts

Subscales “interest in technology in different contexts”	# Items	α	All *M* (*SD*)	Girls *M* (*SD*)	Boys *M* (*SD*)	*t* test (*T* value)	Effect size (*d*)
Interest in technology in leisure time	4	0.78	2.88 (0.69)	2.61 (0.68)	3.13 (0.62)	8.68*	**0.8**
Interest in technology at school	3	0.75	2.60 (0.73)	2.41 (0.66)	2.78 (0.75)	5.70*	0.5
Vocational interest in activities within a design process	7	0.89	2.26 (0.74)	1.87 (0.57)	2.62 (0.69)	13.07**	**1.2**

Cronbach’s alpha of subscales (α); N = 480; mean (M, Likert scale ranging from 1 to 4) and standard deviation (SD) for girls and boys separately; results of *t* test for independent samples (girls and boys); effect size (Cohen’s d): small: 0.2 < *d *< 0.5, medium: 0.5 ≤ *d * < 0.8, large: *d * ≥ 0.8; **p < 0.01, *p < 0.05, *n.s.* not significant. Large effects (*d * > 0.8) are printed in bold type

#### Specific interest in activities within a design process

A comparison between the mean values of the 25 items clearly shows that the theoretical approach to technology (e.g. “Explaining technical contexts to someone,” “Reading about technical topics in newspapers and magazines”) is less interesting for adolescents than hands-on activities (e.g. “Designing something,” “Producing something, building it”). The standard deviations of all items are relatively high, which indicates a broad dispersion of the responses between “Very interested” and “Not interested at all.” With principal component analyses, we were able to extract five factors from a total of 15 items (KMO = 0.92; communalities: *h*^2^ > 0.46; MSA coefficients > 0.86; see “Appendix 2”). These factors explained 61% of the total variance. It is quite noteworthy that the factor “Designing a product in a team” could not be extracted whereas “Designing an eco-friendly product” could be extracted. This implies that technical activities as such determine interest more markedly than their social form.

The mean values of the five scales point to a large extent of interest in inventing, developing, and producing technology (*M* = 2.79) and to a medium extent of interest in using and repairing technology (*M* = 2.59), in designing and planning technology (*M* = 2.57), and in designing eco-friendly products (*M* = 2.57). Interest in understanding and assessing technology is only low, by contrast (*M* = 2.27).

The big mean differences between the five scales with medium to large effects (*d* = 0.3–0.7) confirm the specificity of technology-related interest within the design process. The differences between theoretical and practical activities are quite remarkable (*d* = 0.7). The difference between the scale “Inventing, developing and building” and the scale “Understanding, explaining and evaluating” is particularly large (see Table 2). Moreover, there are significant differences between girls and boys in most scales, with boys being much more interested than girls (*d* ≥ 0.7). Solely the scales “Planning and designing” and “Designing an eco-friendly product” did not reveal gender differences (see Table 2).

**Table 2:** Mean values and gender differences regarding specific interest in design-process activities; sorted from highest to lowest effect size (d)

Subscales of specific interest in design-process activities	# Items	α	All *M* (*SD*)	Girls *M* (*SD*)	Boys *M* (*SD*)	*t* test (*T* value)	Effect size (*d*)
Using and repairing	4	0.80	2.59 (0.78)	2.18 (0.65)	2.97 (0.69)	12.93*	**1.2**
Understanding, explaining and evaluating	5	0.89	2.27 (0.66)	2.04 (0.56)	2.48 (0.66)	7.79*	0.7
Inventing, developing and building	6	0.80	2.79 (0.78)	2.51 (0.74)	3.04 (0.73)	7.87*	0.7
Planning and designing	4	0.75	2.57 (0.71)	2.52 (0.66)	2.62 (0.75)	0.29	n.s.
Designing an eco-friendly product	4	0.71	2.57 (0.72)	2.56 (0.72)	2.58 (0.73)	0.29	n.s.

Cronbach’s alpha of subscales (α); N = 480; mean (M, Likert scale ranging from 1 to 4) and standard deviation (SD) for girls and boys separately; results of *t* test for independent samples (girls and boys); effect size (Cohen’s d): small: 0.2 < *d * < 0.5, medium: 0.5 ≤ *d * < 0.8, large: *d * ≥ 0.8; **p < 0.01, *p < 0.05, *n.s.* not significant. Large effects (*d * > 0.8) are printed in bold type

#### Vocational interest in activities within a design process

Interest in activities concerning the future job varies considerably between girls and boys (*d* > 0.4; see Table 3). Overall, the mean values are even lower than those of the scales presented above. Not even the scale “Designing technical tools” is independent of gender, which is the case if interest is measured without reference to the potential future job (see Tables 1, 2).

**Table 3:** Mean values and gender differences regarding vocational interest in specific activities; sorted from highest to lowest effect size (d)

Single items concerning activities in future job: In my future job, I would like to …	All *M* (*SD*)	Girls *M* (*SD*)	Boys *M* (*SD*)	*t* test (*T* value)	Effect size (*d*)
Construct and build technical tools	2.12 (0.98)	1.66 (0.71)	2.56 (0.98)	11.59**	**1.1**
Invent and develop new technical tools	2.11 (0.94)	1.67 (0.72)	2.51 (0.95)	10.98**	**1.0**
Explain and sell technical tools	2.18 (0.88)	1.78 (0.69)	2.56 (0.87)	10.74**	**1.0**
Know more about technical tools	2.28 (0.93)	1.87 (0.75)	2.67 (0.91)	10.47**	**1.0**
Use technical tools	2.58 (0.96)	2.13 (0.88)	2.95 (0.86)	10.10**	**0.9**
Develop new tools in a team	2.23 (0.92)	1.83 (0.72)	2.59 (0.94)	10.06**	**0.9**
Deal with people	3.13 (0.81)	3.39 (0.70)	2.89 (0.83)	7.17**	0.7
Design technical tools	2.32 (0.98)	2.14 (0.92)	2.49 (0.99)	4.02**	0.4

N = 480; mean (M, Likert scale ranging from 1 to 4) and standard deviation (SD) for girls and boys separately; results of *t* test for independent samples (girls and boys); effect size (Cohen’s d): small: 0.2 < *d * < 0.5; medium: 0.5 ≤ *d * < 0.8; large: *d * ≥ 0.8; **p < 0.01; *p < 0.05, *n.s.* not significant. Large effects (*d * > 0.8) are printed in bold type

The items “Inventing and developing in future job” and “Constructing and building in future job” have the lowest mean (*M* = 2.12 and 2.11) within the scale of vocational interest (Table 3). In the non-occupational context of the scale that relates to specific interest in design activities, the same items received much higher ratings (*M* = 2.79, see Table 2). Furthermore, there are again marked differences between girls and boys (*d* > 1.0). The item “Using technical tools in the future job” shows the highest mean of all items that explicitly relate to technical devices or technology-specific activities (*M* = 2.58). The only activity that is not specific to technology, namely “Dealing with people in the future job,” received even higher ratings, however (*M* = 3.13), with girls expressing much more interest than boys.

### Research question 2: self-efficacy

The following section presents the results for research question 2: *How does self*-*efficacy in technical tasks as perceived by girls and boys differ between different tasks (irrespective of the content) within the design process?*

The average of a total of 24 items relating to perceived technology-specific self-efficacy ranges from 2.33 (“Even if I am under time pressure, I can draw very accurate sketches and plans”) to 3.06 (“When I select a technical device, I always know what is important to me”). By means of principal component analyses, we could extract four factors from a total of 15 items (KMO = 0.89; MSA coefficients > 0.81; see “Appendix 3”). These factors explained 60% of the total variance. The mean values of the scales point to a medium perception of technology-specific self-efficacy (2.53 < *M* > 2.72). Overall, “Using and repairing” (*M* = 2.57) and “Planning and designing” received the lowest ratings (*M* = 2.52). In contrast to interest, self-efficacy is most pronounced with respect to the scale “Understanding and explaining” (*M* = 2.72). The differences between the scales are not large, however (*d* = 0.2–0.3).

In most technical activities, the girls perceived their self-efficacy much more negative than the boys, particularly regarding the category “Using and repairing” (*d* = 1.3; see Table 4). In this area, girls do not feel confident at all while they reported the same extent of self-efficacy as the boys regarding the category “Planning and designing”. These are also those activities that interest girls the most (see Table 1). Accordingly, the correlations between interest and self-efficacy in design processes proved to be high (*r* = 0.6).

**Table 4:** Mean values and gender differences in perceived self-efficacy regarding technical tasks; sorted from highest to lowest effect size (d)

Subscales of perceived self-efficacy in technical tasks	# Items	α	All *M* (*SD*)	Girls *M* (*SD*)	Boys *M* (*SD*)	*t* test (*T* value)	Effect size (*d*)
Using and repairing	6	0.88	2.57 (0.83)	2.09 (0.66)	3.01 (0.72)	14.57**	**1.3**
Understanding and explaining	8	0.82	2.72 (0.57)	2.49 (0.50)	2.94 (0.55)	9.17**	**0.8**
Planning and designing	3	0.80	2.52 (0.81)	2.44 (0.82)	2.60 (0.80)	2.21	n.s.
Craft-related tasks	2	0.78	2.73 (0.82)	2.55 (0.82)	2.89 (0.79)	4.63**	0.4

Cronbach’s alpha of subscales (α); N = 480; mean (M, Likert scale ranging from 1 to 4) and standard deviation (SD) for girls and boys separately; results of *t* test for independent samples (girls and boys); effect size (Cohen’s *d *): small: 0.2 < *d * < 0.5, medium: 0.5 ≤ *d * < 0.8, large: *d *  ≥ 0.8; **p < 0.01, *p < 0.05, *n.s.* not significant. Large effects (*d * > 0.8) are printed in bold type

### Research question 3: structural equation model

The following section presents the results for research question 3: *Can vocational interest in the field of technology and design be predicted by interest in technology and self*-*efficacy in technical tasks?*

This question was answered on the basis of a structural equation model (see Fig. 3) that related to whether interest in dealing with technology in leisure time (i.e. subjective task value) or technology-specific self-efficacy (i.e. expectations of success) more strongly influences vocational interest (i.e. achievement-related choices/interest; see Fig. 1). The missings in the data of the total sample (approximately 2–5 per item or scale) were replaced by mean values because the omission of all cases with a missing in the 13 items or scales would have resulted in a considerable reduction (approximately *N* = 50) of the sample size.

If the model is calculated with the total sample (*N* = 483), the latent variables are well represented by the manifest variables with standardized weights or a factor loading of 0.50–0.96 and variances explained by the latent variables of 0.25–0.92. The fit indices of the model can be assigned to the good or even very good range, which applies to the overall sample as well as to the two groups separately (see Table 5). The fit indices alone do not say much about the quality of the model, however. An assessment of the quality requires a detailed analysis of the assumed relationships between and the influences of the latent variables.

**Table 5:** Assessment criteria and fit values for the structural equation model

Fit-indices	Fit criteria		Fit values of model sample N = 483	Fit values of model multigroup analysis girls N = 230/boys N = 253
	Good	Unfit		
CMIN	–	–	96	158
CMIN/DF	~ 1	> 5	1.66	1.38
CFI	> 0.95	= 0	0.99	0.98
TLI	> 0.95	= 0	0.98	0.98
SRMR	< 0.11	1	0.033	0.052
RMSEA	< 0.05	> 0.1	0.037	0.028

The limits of the quality areas vary in part between Airbuckle (2007), Byrne (2001) and Hair et al. (1998)

**Fig. 4:**
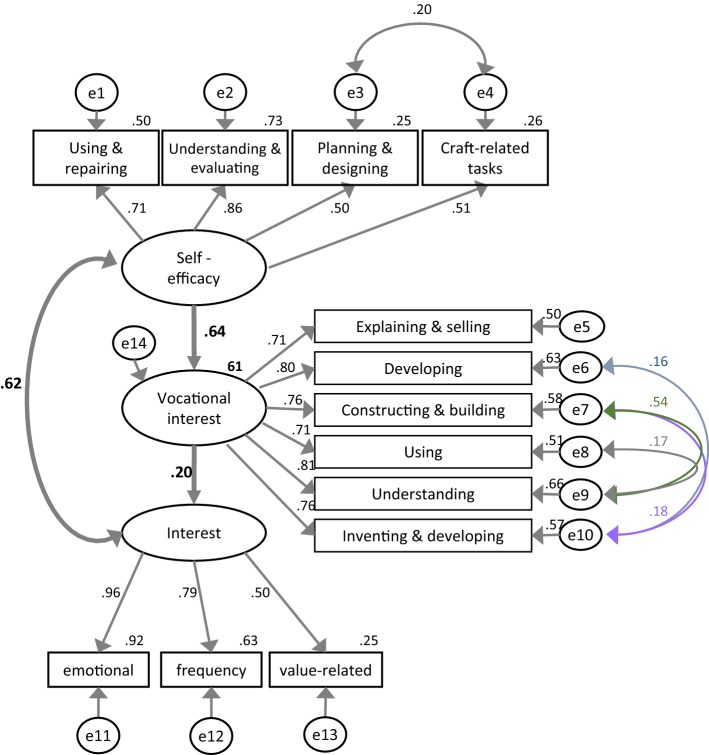
Structural equation model with self-efficacy (SE) and interest as independent (endogenous) variables and vocational interest as a dependent (exogenous) variable. Model fitted with the data of the total sample (N = 483). Standardized coefficients; measured variables are represented in boxes, latent variables in ovals

The size of the standardized coefficients (see Fig. 4) suggests that the theoretically assumed relationships between the latent variables in the overall model are all relevant. Self-efficacy and interest can predict vocational interest in the overall model with 61% explained variance. The path coefficient (regression coefficient) between self-efficacy and vocational interest (0.64) is three times higher than the path coefficient between interest and vocational interest (0.20). The correlation between the two latent variables (“Interest” and “Self-efficacy”) is high (0.62). Owing to the marked content-related connections between some manifest scales or items, covariances between the associated errors are to be assumed. This applies to errors concerning the self-efficacy scales “Planning and designing” and “Craft-related tasks” as well as five items characterizing vocational interest. Direct correlations between manifest variables are not possible.

**Fig. 5:**
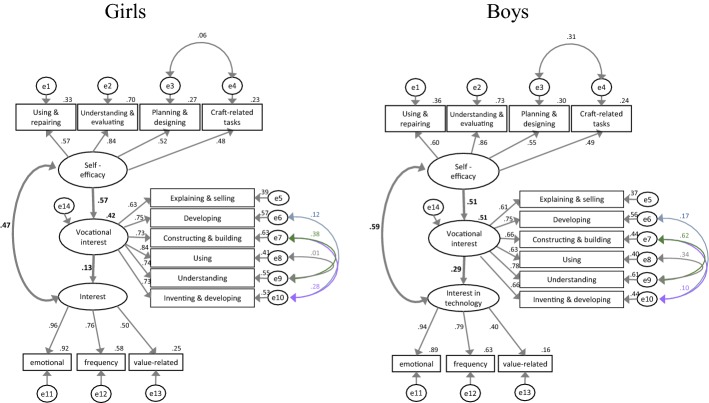
Group comparison between girls and boys with self-efficacy and interest as independent (endogenous) variables and vocational interest as a dependent (exogenous) variable. Model fitted with the data of the total sample in the pretest (N = 483). Standardized coefficients; measured variables are represented in boxes, latent variables in ovals

A group comparison (multigroup analysis following Airbuckle 2007) between girls and boys revealed that the model is valid for both groups (see Table 5). Total explained variance in vocational interest turned out to be smaller than in the total sample (girls: 43%, boys: 51%). The path coefficients connecting “Interest in technology in leisure time” and “Vocational interest in technology” differ significantly between the two groups: in the case of the girls, interest has a weaker influence on vocational interest (0.13) than in the case of the boys (0.29, see Fig. 5) and is not significant.

## Discussion and conclusion

In this section, we first discuss the results of our analysis and thereafter relate them to the current situation and to educational developments in technology education both globally and particularly in Switzerland.

### Gender stereotypes

The data mirrors the traditional stereotypical picture: the boys in our sample are clearly more interested in technology than the girls, especially in the context of the future job. Thus, looking at technology-specific interest in more detail did not change the common overall picture. Although there are some activities that attracted the girls’ interest so that the gender gap did not become manifest (“Planning and designing” and “Designing eco-friendly products”), it seems in general that as soon as the word “technical” occurs, girls feel less attracted than boys. With respect to the design of the study this could indicate that the results might have looked differently if the formulation of the items had avoided “technical” altogether and if the word had been replaced by alternatives like “devices” or “products.” What is nevertheless surprising is that both girls and boys felt much less attracted by “Inventing, developing, constructing and building” in connection with the future job than without mention of a specific context. Apparently, being actively involved in technical activities—in crafts, for instance—is considered to be fun, but still adolescents cannot imagine occupying themselves with such activities on a professional basis. One explanation for this finding could be that these activities are perceived as something very complex and difficult in the real “high-tech job world” so that only brains who studied electro engineering or informatics can cope with them. This assumption is not completely unfounded in the digital age but nevertheless somewhat alarming because the next generation (of the industrialized world) is not willing to take responsibility for current technological developments although they affect our (social) lives more than ever before. Another likely interpretation of this finding is that the “professional world” in general is something rather distant at this age, that is to say 1 or 2 years before career choice. This explanation points to the importance establishing real contacts between the professional world and school already right from the beginning of lower secondary-level schooling, for example by offering short internships, regular opportunities to gain some practical experience, insights into different job profiles, or excursions.

The results concerning the gender gap in terms of the participants’ interest in different activities in the future job indicate that girls seem to have quite well-defined ideas of the contexts in which they wish “to deal with people.” As our analyses show, they do not want to do so in a technical context, because otherwise their ratings of the items “Selling technical tools” or “Developing new tools in a team” would have been much more positive. This low extent of interest in technical activities irrespective of the activity as such (selling, explaining, designing, inventing, etc.) suggests that the vocational interests of girls are more strongly shaped by the context “future job” or by the working sector than by the concrete activity itself. Besides, we know from pertinent literature that in the eyes of females, the image of technical professions does not correspond with the notion of an ideal profession (see acatech and VDI 2009).

### Significance of self-efficacy

As our analyses have shown, self-efficacy is a crucial factor of self-assessments of technology related abilities, which is consistent with Bandura’s (1997) work on self-efficacy beliefs in learning. The findings of our study are obvious, especially with respect to gender differences, and raise the following question: how can the considerable differences between girls and boys that became apparent in the structural equation model be accounted for?

Overall, the simplified expectancy-value model (Fig. 1) based on Eccles and Wigfield (2002) could be empirically confirmed. This noteworthy insofar subfactors and subvariables had not been incorporated into our version of the model. The subjective value of the task, for example, was represented by only one scale, namely “Interest in the context of leisure time,” which includes the emotional and value-related facets of interest (see Krapp and Prenzel 2011) as well as the frequency of the activity. Thus, compared to the “task value” considered by Eccles and Wigfield (2002), the “cost” variable (opportunity costs) was missing. It may be connected with the frequency of activity, however. The factor “Expectations of success” was comprehensively integrated through the four scales covering technology-specific self-efficacy (see Fig. 3). The analysis of the entire sample pointed to an unequal influence of interest and self-efficacy on vocational interest. If, however, the model was examined for the subgroup of the girls, the model fit got worse so that the prediction of vocational interest was no longer satisfactory. This indicates that apart from interest and self-efficacy other variables, which have not been included in the model so far, may influence the vocational interests of girls. According to Eccles and Wigfield (2002), variables like cultural milieu, perceptions, goals, self-schemata, previous achievements, affective reactions, and memories have an indirect effect on achievement-related choices. As for girls, it seems likely that certain social factors also have direct implications. Still, the path coefficient between self-efficacy and vocational interest is comparatively high (0.57) whereas the path coefficient between interest and vocational interest disappeared almost completely (0.13). From this we can conclude that girls tend to rule out a technical career for emotional or value-related reasons that are in line with their expectations of success in solving technical problems.

These findings lead to the following fundamental question: how can technology-specific self-efficacy be built up and promoted? Bandura (1997), the originator of the concept of self-efficacy, discussed four sources of self-efficacy: (1) mastering difficult situations; (2) observation of models; (3) social support; (4) perception of one’s own emotional excitement. If individuals do not experience these sources, they can hardly develop feelings of self-efficacy. By linking this theory and our results to the usual teaching practices in the science and/or technical design classroom, we can provide teachers with information on how important it is to foster the four sources of self-efficacy especially in girls. Another way of increasing feelings of self-efficacy and thus of encouraging interest in technology consists in providing technology education on a long-term basis. Acatech and VDI (2009), for instance, were able to show a positive effect of continuous technical teaching in Germany: “If the students are offered adequate technology education—as in some of Germany’s federal states—interest in technology is … higher” (p. 30, own translation). MoMoTech (Acatech 2011) even states that promoting technology at school alone is not enough and consequently recommends establishing networks between schools and out-of-school facilities. For achieving sustainable effects, such initiatives should ideally already include kindergarten and preschool. Starting with technology-related classes and programs in lower secondary school is too late. According to Erikson (1982), middle childhood is a particularly influential time for interventions because this is when behavioral habits that are critical to health and competence become entrenched and set and when skills that form the basis for personal identities and self-esteem are acquired. Furthermore, Simpkins et al. (2006) found that individual differences in self-beliefs and task beliefs develop in the course of the elementary school years and then are steadily being refined in response to feedback on performance and in processes of identity formation during adolescence. Against this background, it seems to be highly advisable or even vital to introduce multidimensional technology education already in elementary school, that is to say at the age when self-beliefs and task beliefs start to develop.

### Attracting young people by combining science and design

As many studies have shown and teachers know, it is quite hard to arouse young people’s interest in technology via theoretical approaches. Therefore, alternative ways of accessing the world of technology need to be looked for. Our results indicate that integrating practical, theoretical, creative, and social aspects of technology can make the subject attractive to many adolescents. By including product design as one part of technology education, several aspects can be connected, thus offering students a unique and special learning experience. In our study, we could observe that building cognitive bridges between practical work (manual skills) and theoretical contexts (knowledge of science) poses a great challenge for teachers as well as for students (Guedel 2014). Thus, one central question is how it is possible to bring these different activities (practical, theoretical, creative, social) together in such a way that it is not only fun, but the students also learn something? And what can they learn?

Lewis et al. (1998) suggested that the focus of technology teaching needs to shift from a design process or problem-solving approach to an approach that fosters problem posing. This approach is especially interesting to design teachers. Among the advantages Lewis et al. (1998) see the promotion of active participation and an increased likelihood that the teaching is characterized by authenticity and creativity. As children need to understand the situation from which the problem arises (Lewis et al. 1998), they should be allowed enough time to immerse themselves in the context of the task before the specific need, opportunity, or problem is presented or addressed. Further suggestions for successful learning in technology education have been proposed by Mawson (2003, p. 123): “The task itself should be sufficiently open to provide the possibility of a range of solutions being developed and the children would be encouraged to choose a starting strategy which fitted their own preferred learning styles. As designing and making occur concurrently, at regular intervals children should be required to explain/show, and discuss their developing solutions.”

Another approach, which is of special interest particularly to science teachers, has been outlined by Kim and Roth (2016, p. 153): “To integrate the societal emphasis on engineering and technology in the school curriculum, science teachers are encouraged to integrate in their curricula engineering and technological design tasks such as designing and building structures (i.e., boats, bridges, or devices).” This is supported by a meta-analysis on context-based science education, showing very large effects for societal contexts (STS = Science and technology in Society; Bennett et al. 2007).

Such recommendations often rest on those dimensions that science and technology share rather than on those dimensions in which they differ (Roth 2001). This is why there is a tendency to prioritize science content knowledge and skills acquired in hands-on technological design (Dohn 2013). In other words, within the framework of this approach, teachers regard classroom activities that relate to technology and engineering as a means for extending their students’ knowledge of science and accordingly tend to focus on how certain scientific concepts can be applied and verified in technological design. Kim and Roth (2016, p. 153) draw a similar conclusion: “There has been much emphasis on enhancing students’ science content knowledge and the application of science in technology and engineering education.” Although such efforts are to be welcomed in principle and can be regarded as a first step towards the aim of making students familiar with the world of technology, they are not sufficient to do justice to technology as a distinct domain that is worth being dealt with for its own sake and not just as a subordinate or “junior” field of the natural sciences. Hence, Kim and Roth (2016, p. 153) add: “…, it is important to remember the different goals of the two disciplines, and that they are both depending on each-other but have different goals.”

## Conclusion

The results presented in this contribution might provide useful indications how technology interest, self-efficacy and vocational interest could be enhanced in the pivotal age group in secondary level I, in particular being aware of the gender differences.

Combining practical, theoretical, creative, and social aspects of technology in the classroom could enhance the attractiveness of technology related subjects. One research based way for this would be to include product design as one part of technology education. It should be kept in mind, however, that building cognitive bridges between practical work (manual skills) and theoretical contexts (knowledge) pose a great challenge, which makes professional assistance, based on existing research, indispensable. Teachers should be aware how much influence they can have with specific settings and activities and with their own behaviour on the self-efficacy beliefs of the pupils, especially for strengthening the self-efficacy of girls regarding their specific interests and approaches.

School boards and curriculum planners must recognize how important it is to offer possibilities for approaches to technology teaching, especially in integrative approaches combined with science teaching and by offering manifold contacts to the technical world and by giving opportunities to practical activities within a design process.

And last but not least educational research and teacher education should focus on new and subject specific approaches to technology education on all levels, and encourage teachers to integrate technology education into their programs and to seek contacts to the professional world. Finally, and probably most important, teacher education should take care to enhance their proper technology specific self-efficacy, in order to enhanceself-efficacy of their students.

## Appendix 1: Principal component analysis: general and specific interest in the contexts of school, leisure time, and future job

In order to reduce the data and to form interpretable item groups, a principal component analysis was carried out on the 15 interest items related to school, leisure time, and future job. (without Iallg_S01 and Iallg_B01) (see Bühner 2006, pp. 207). One item (Iallg_S05) had not loaded highly on any factor and was therefore omitted in a second principal component analysis. The principal component analysis with Promax rotation extracted three factors from the item pool (15 items) (see Table 6). There were substantial correlations that justified a principal component analysis (KMO = 0.89). The determination of the communalities refers to the extraction of three factors. The proportion of variance between the items that is explained by the three factors is larger than 45% (*h*^2^ > 0.45). The eigenvalue indicated that a 3-factorial analysis was appropriate according to the eigenvalue criterion > 1. Three factors together account for 64% of the total variance. The correlations of the three factors are significant with > 0.46. A Promax rotation was mandatory in this case (> 0.1) (Bühner 2006). The factors and thus the three contexts under investigation proved to be not independent of each other.

**Table 6:** Structural matrix of the principal component analysis of the items related to school, leisure time, and future job. Three factors are extracted: Ispezt_B, Iallg_S, and Iallg_F

Items	Factor 1: Vocational interest in activities within a design process (Ispezt_B)	Factor 2: Interest in technology at school (Iallg_S)	Factor 3: Interest in technology in leisure time (Iallg_F)
Ispezt_B07	0.97		
Ispezt_B04	0.94		
Ispezt_B02	0.82		
Ispezt_B06	0.70		
Ispezt_B03	0.69		
Ispezt_B01	0.67		
Ispezt_B05	0.62		
Iallg_S03		0.88	
Iallg_S04		0.87	
Iallg_S02		0.72	
Iallg_F03			0.89
Iallg_F04			0.84
Iallg_F01			0.61
Iallg_F02			0.59

KMO = 0.89; Communalities: h^2^ > 0.45; MSA coefficients > 0.84. Three factors explain 64% of the total variance. Factor loadings < 0.4 are not displayed

The three factors can be assigned to the three factors pertaining to the contexts “career” (7 items), “school” (3 items) and “leisure time” (4 items). The result of the principal component analysis thus shows that the items have a structure that is comprehensible in terms of content and that the interest in technology is specific to a particular sector of life. This means that someone expressing a high extent of interest in technology in leisure time is not necessarily highly interested in technology at work. According to Bortz (2006), factors 1 and 3 fulfill the requirements for well-defined subscales: at least 4 items with loads higher than 0.6. Factor 2 does not meet these criteria, but it has a good reliability with α = 0.75 and was therefore accepted as a scale. The 14 items could thus be summarized in three subscales: “Interest in technology in leisure time,” “Interest in technology at school,” and “Interest in technical activities in the future job.”

## Appendix 2: Principal component analysis: specific interest in activities within the design process

As for the interest dimension discussed in app. A, a principal component analysis was carried out on the 25 items related to the design process. The items V_B06 and UMW03 had not loaded highly on any factor, which may have something to do with the formulation of the items. The two items were therefore omitted in the second principal component analysis. The principal component analysis with Promax rotation extracted five factors from the item pool (23 items) (see Table 7). There were substantial correlations that justified a principal component analysis (KMO = 0.92). The determination of the communalities referred to the extraction of five factors. The communalities of the items are all sufficiently high (*h*^2^ > 0.46). This means that the items are sufficiently covered by the five factors. The proportion of variance between the items that is explained by the five factors is larger than 46%. The eigenvalue indicated that a 5-factorial analysis was appropriate according to the eigenvalue criterion > 1. Five factors together account for 61% of the total variance. The correlation of the five factors is significant with > 0.18. Promax rotation is mandatory in this case (> 0.1). The factors and thus the categories of technical activities are not independent of each other.

**Table 7:** Structural matrix of the principal component analysis of items of specific interest in activities within the design process. Five factors are extracted: Ispezt_ENTW, Ispezt_NU_H, Ispezt_V_B, Ispezt_UMW, Ispezt_SKIZ

Items	Factor 1: Inventing, developing and building (Ispezt_ENTW)	Factor 2: Using and repairing (Ispezt_NU)	Factor 3: Understanding, explaining and evaluating (Ispezt_V_B)	Factor 4: Designing eco-friendly products (Ispezt_UMW)	Factor 5: Planning and designing (Ispezt_SKIZ)
Ispezt_ENTW_06T	0.81				
Ispezt_ENTW_05	0.75				
Ispezt_ENTW_04T	0.67				
Ispezt_ENTW_03	0.66	0.41			
ispezt_ENTW_01T	0.62				
Ispezt_ENTW_02	0.62				
Ispezt_SKIZ_03T	0.48				0.36
Ispezt_SKIZ_02	0.48				0.39
Ispezt_NU_02		0.75			
Ispezt_NU_03	− 0.32	0.75			
Ispezt_NU_04		0.66			
Ispezt_NU_01		0.64			
Ispezt_V_B_03			0.80		
Ispezt_V_B_04T			0.78		
Ispezt_V_B_01			0.71		
Ispezt_V_B_02			0.59		
Ispezt_V_B_05			0.55		
Ispezt_UMW_01				0.79	
Ispezt_UMW_04	0.35			0.71	
Ispezt_UMW_05	0.37			0.66	
Ispezt_UMW_02				0.58	
Ispezt_SKIZ_04					0.86
Ispezt_SKIZ_01					0.78

KMO = 0.92; communalities: h^2^ > 0.46; MSA coefficients > 0.86; 5 factors together explain 61% of the total variance. Factor loadings < 0.3 are not displayed

The five factors can largely be assigned to the five theoretically derived categories. Only the social form “Teamwork” (ending “T”) was not represented in the principal component analysis, and the category SKIZ was not clearly represented. The items with team formulations can be found almost exclusively in Factor 1. Thus, technical activities determine interest more than the social form.

According to Bortz (2006), Factors 1 and 2 are sufficiently well-defined for a subscale: at least 4 items with loadings higher than 0.6. Factors 3 and 4 almost met the requirements and were therefore included as subscales in the following analyzes. Strikingly, the negative loading of NU03 (“Watching someone working”) belongs to Factor 1 that related to actively inventing, developing, and manufacturing technology. Factor 2 also includes the passive use of technology. Factor 5 does not meet the requirements. With only two items, it is not possible to build a subscale. The two items SKIZ_03T and SKIZ_02 have a loading higher than 0.36 on factor 1. A scale with the four SKIZ items had a sufficiently high reliability (α = 0.71) and was also accepted as a scale. The 23 items could therefore be summarized in five subscales: “Inventing, developing and producing interest in technology” (Ispezt_ENTW), “Using interest in technology” (Ispezt_NU), “Understanding and assessing interest in technology” (Ispezt_V_B), “Interest in technology with environmental relevance” (Ispezt_UMW), and “Designing, organizing and shaping interest in technology” (Ispezt_SKIZ).

## Appendix 3: Principal component analysis: technology-specific self-efficacy

Finally, a principal component analysis was also carried out on the 24 items related to technology-specific self-efficacy. The items NU_04, PL_02, WE_02, TEAM, and WE04 did not load highly on any factor, which could be put down to, inter alia, the negative formulation of the items and to the fact that they are items that represent a group (e.g., TEAM) in content. The five items were excluded from the second principal component analysis (see Table 8).

**Table 8:** Structural matrix of the principal component analysis of the items related to technology-specific self-efficacy. Five factors are extracted: SWEspez_NU, SWEspez_VE, SWEspez, SKIZ, SWEspez_WE

Items	Factor 1: Using and repairing (SWEspez_NU)	Factor 2: Understanding and explaining (SWEspez_VE)	Factor 3: Planning and designing (SWEspez_SKIZ)	Factor 4: Craft-related tasks (SWEspez_WE)
SWEspez_NU_01	0.83			
SWEspez_NU_03	0.82			
SWEspez_NU_06	0.81			
SWEspez_NU_07	0.79			
SWEspez_NU_02	0.74			
SWEspez_NU_05	0.59			
SWEspez_VB_05		0.86		-0.32
SWEspez_VB_04		0.81		
SWEspez_VB_01		0.74		
SWEspz_VB_02		0.64		
SWEspez_VB_03		0.61		
SWEspez_PL_03		0.51		
SWEspez_PL_01		0.47		
SWEspez_PL_04		0.31		
SWEspez_SKIZ01			0.86	
SWEspez_SKIZ02			0.85	
SWEspez_SKIZ03			0.77	
SWEspez_WE_03				0.87
SWEspez_WE_01				0.85

KMO = 0.89; Communalities: h^2^ > 0.28 (PL_04); MSA coefficients > 0.81 (WE_03). Four factors together explain 60% of the total variance. Factor loadings < 0.3 are not displayed

The principal component analysis with Promax rotation extracted four factors from the item pool (19 items) (see Table 8). There were substantial correlations that justified a factor analysis (KMO = 0.89). The identification of the communalities related to the extraction of four factors. Except for one item, the communalities are sufficiently high (*h*^2^> 0.38). This means that the items are sufficiently covered by the five factors. The proportion of variance between the items that is explained by the five factors is larger than 38%. The eigenvalue indicated that a 4-factorial analysis was appropriate according to the eigenvalue criterion > 1. Four factors together account for 60% of the total variance. The correlations of the four factors are significant with > 0.34. Promax rotation is mandatory in this case (> 0.1). The factors and thus the categories of technical activities are not independent of each other.

For the most part, the four factors can be assigned to the four theoretically derived categories. Only the category “Problem Solving” (PL) is not represented as an independent factor. The three items are contained in the factor VE (“Understanding and Explaining”). This is comprehensible in terms of content, since the items all address theoretical problem solving. The result of the principal component analysis thus shows that the items have a structure that is comprehensible in terms of content.

According to Bortz (2006), Factors 1 and 2 are sufficiently well-defined for a subscale: at least 4 items with loads greater than 0.6. Factors 3 and 4 almost met the requirements and were therefore included as subscales in the following analyzes. What was striking is the negative loading of VE_05 (“In conversations about technical progress, I have no inhibitions to express my opinion”) on Factor 4 that contains the two items relating work instruction. Self-efficacy expectations regarding discussions about technology have thus only little to do with the expectation of self-efficacy in work instruction. Factor 1 refers to the active invention, development, and manufacture of technology.
